# Protein Malnutrition Induces Bone Marrow Mesenchymal Stem Cells Commitment to Adipogenic Differentiation Leading to Hematopoietic Failure

**DOI:** 10.1371/journal.pone.0058872

**Published:** 2013-03-14

**Authors:** Mayara Caldas Ramos Cunha, Fabiana da Silva Lima, Marco Aurélio Ramirez Vinolo, Araceli Hastreiter, Rui Curi, Primavera Borelli, Ricardo Ambrósio Fock

**Affiliations:** 1 Department of Clinical and Toxicological Analyses, Faculty of Pharmaceutical Sciences, University of São Paulo, São Paulo, Brazil; 2 Department of Genetics, Evolution and Bioagents. Institute of Biology, University of Campinas-Unicamp, Campinas, Brazil; 3 Department of Physiology and Biophysics, Institute of Biomedical Sciences, University of São Paulo, São Paulo, Brazil; Georgia Regents University, United States of America

## Abstract

Protein malnutrition (PM) results in pathological changes that are associated with peripheral leukopenia, bone marrow (BM) hypoplasia and alterations in the BM microenvironment leading to hematopoietic failure; however, the mechanisms involved are poorly understood. In this context, the BM mesenchymal stem cells (MSCs) are cells intimately related to the formation of the BM microenvironment, and their differentiation into adipocytes is important because adipocytes are cells that have the capability to negatively modulate hematopoiesis. Two-month-old male Balb/c mice were subjected to protein-energy malnutrition with a low-protein diet containing 2% protein, whereas control animals were fed a diet containing 12% protein. The hematopoietic parameters and the expression of CD45 and CD117 positive cells in the BM were evaluated. MSCs were isolated from BM, and their capability to produce SCF, IL-3, G-CSF and GM-CSF were analyzed. The expression of PPAR-γ and C/EBP-α as well as the expression of PPAR-γ and SREBP mRNAs were evaluated in MSCs together with their capability to differentiate into adipocytes *in vitro*. The malnourished animals had anemia and leukopenia as well as spleen and bone marrow hypoplasia and a reduction in the expression of CD45 and CD117 positive cells from BM. The MSCs of the malnourished mice presented an increased capability to produce SCF and reduced production of G-CSF and GM-CSF. The MSCs from the malnourished animals showed increased expression of PPAR-γ protein and PPAR-γ mRNA associated with an increased capability to differentiate into adipocytes. The alterations found in the malnourished animals allowed us to conclude that malnutrition committed MSC differentiation leading to adipocyte decision and compromised their capacity for cytokine production, contributing to an impaired hematopoietic microenvironment and inducing the bone marrow failure commonly observed in protein malnutrition states.

## Introduction

Protein malnutrition decreases the production of blood cells, leading to bone marrow hypoplasia and inducing structural alterations interfering with both innate and adaptive immunity [1], [2], [3], [4]. Blood cells arise in the bone marrow from stem cells able to undergo processes of proliferation and differentiation in the hematopoietic microenvironment [5], [6].

Adequate hematopoiesis is dependent on an intact and functional bone marrow microenvironment, which is an environment fully competent to provide the appropriate signals through the production of soluble factors and cell-cell contact interactions regulating by several mechanisms, directly or indirectly, the self-renewal, proliferation, survival, migration and differentiation of hematopoietic cells [6], [7], [8].

The cellular constituents of the bone marrow microenvironment, also defined as the hemopoietic niche, largely derive from a common progenitor of mesenchymal origin called mesenchymal stem cells (MSCs), initially identified in adult bone marrow and that have classically the ability to self-renew and differentiate into tissues of mesodermal origin such as osteocytes, chondrocytes and adipocytes [6], [7].

The bone marrow adipocyte is one type of cell that currently has been paid more attention for being able to modulate hematopoiesis and that is not just a type of tissue filler [8], [9]. The literature reports on the importance of the bone marrow adipocytes in complex hematopoietic regulation and on the influence of bone marrow adipocytes as negative regulators of hematopoiesis compromising the capacity to produce growth factors such as granulocyte-macrophage colony stimulator factor (GM-CSF) and granulocyte colony stimulator factor (G-CSF) [8].

In the bone marrow, the differentiation and maturation of mesenchymal stem cells are harmoniously controlled by specific signal transduction and specific groups of transcription factors. In this context, adipogenic differentiation is controlled by different transcriptional factors, such as members of the CCAAT/enhancer-binding protein (C/EBP) family, like C/EBPα and C/EBPβ, that initiate the differentiation of adipocytes followed by the activation of the peroxisome proliferator-activated receptor γ (PPAR-γ), a member of the nuclear hormone receptor family that regulates adipocyte-specific gene expression and is critical for the maturation of lipid-filled adipose cells from pluripotent stem cells through the sterol regulatory element binding proteins (SREBPs) that regulate lipid homeostasis [10], [11]. Attention is given to PPAR-γ, which in isolation can initiate adipogenic differentiation without inducers, and C/EBPα, a second transcription factor induced during adipocyte differentiation, that cooperates with PPAR-γ to dramatically stimulate the adipocyte program [12].

Knowing that hematopoietic tissues, like all tissues that have a high rate of cell renewal and proliferation, require an ample supply of nutrients and may thus be altered by deficient states, our group demonstrated that in the situation of protein malnutrition, hematopoietic commitment with reduction of hematopoietic stem cell proliferation committing granulopoiesis, which contributes to the peripheral leukopenia and neutropenia commonly found in protein malnutrition states, suggesting a state of bone marrow failure [1], [2], [3], [13]. In this study, we decided to investigate the influence of protein malnutrition on modifying the bone marrow mesenchymal stem cells and on the formation of adipocytes that influence hematopoiesis.

## Materials and Methods

### Diets

The murine diets were prepared in our laboratory. The mineral and vitamin mixtures were prepared according to the recommendations of the American Institute of Nutrition for adult mice [14]. Casein (>85% protein) was used as the protein source. Both diets contained 100 g/kg sucrose, 80 g/kg soybean oil, 10 g/kg fiber, 2.5 g/kg choline bitartrate, 1.5 g/kg L-methionine, 40 g/kg of the mineral mixture and 10 g/kg of the vitamin mixture. The control diet contained 120 g/kg casein and 636 g/kg cornstarch, while the malnourishment diet contained 20 g/kg casein and 736 g/kg cornstarch. Except for the protein content, the two diets were identical and isocaloric; the total amount of casein removed from the formulation of the hypoproteic diet was substituted by the same mass of cornstarch. The diets were isocaloric and provided 1716.3 kJ/100 g. The final protein content was confirmed using the standard micro-Kjeldahl method [15].

### Animals

Two-month-old, male inbred BALB/c mice were obtained from the animal laboratory at the Faculty of Pharmaceutical Sciences at the University of São Paulo**.** The mice were placed in individual “metabolic cages” and received the control diet for 7 days until their body weight stabilized. After this period of adaptation, the animals were divided into two groups and maintained under a regular 12 h light/dark cycle at a temperature of 22–25°C and a relative humidity of 55±10%, receiving either the control or the low-protein diet and water *ad libitum.* Body weight and food consumption were monitored every 48 h. The mice were subjected to experimental assays after 21 days of eating their respective diets, when members of the malnourished group had lost approximately 20% of their original body weight. A nutritional evaluation was performed by measuring the body weight and diet consumption, the protein, albumin and pre-albumin concentrations and the hematological parameters. The body weight variation was calculated using the initial (after the adaptation period) and final weight (day of sacrifice) of the animals in both of the groups, and the results are expressed as the mean plus or minus the standard deviation. This study was approved by the Ethics Committee of the Faculty of Pharmaceutical Sciences at the University of São Paulo (protocol number 277/2010), in accordance to the guidelines of the Brazilian College on Animal Experimentation. All efforts were made to minimize animal suffering and to reduce the number of animals used.

### Blood

The mice from the control and malnourished groups were anesthetised with xylazine chlorohydrate (Rompum^®^, 10 mg/kg, Bayer S.A., São Paulo, SP, Brazil) and ketamide chlorohydrate (Ketamina^®^, 100 mg/kg, Cristália Ltd., Itapira, SP, Brazil), and then, whole blood samples with and without EDTA (1 mg/mL) were obtained via cardiac puncture. After the blood collection, the anesthetized animals were sacrificed. The hemogram parameters were determined by automatic methods using an ABC Vet instrument (Horiba ***ABX*** Diagnostics, ***Montpellier***, France). The differential leukocyte counts were performed on blood smears stained with the standard May-Grunwald Giemsa solutions (Sigma Chemical Co., St. Louis, MO, USA). The serum was separated by centrifugation; the content of total protein, albumin, and pre-albumin was monitored by the standard methods used in medical analyses.

### Spleen Cellularity

The spleens of the control and malnourished mice were removed, placed in Petri dishes containing 5 mL of Dulbecco’s modified Eagle’s ***medium*** (DMEM) (Vitrocell, Campinas, SP, Brazil) with EDTA (1 mg/mL) and dissociated gently using needles and tweezers. Total cells were determined using a Neubauer chamber and the differential cell counts were performed on smears stained with the standard May-Grünwald Giemsa solutions (Sigma Chemical Company, St. Louis, MO, USA).

### Bone Marrow Histology

Mice from the control and malnourished groups had the sternum removed, which was immediately immersed in a 4% paraformaldehyde fixative at room temperature for 24 h. The sternums were decalcified in 5% EDTA (pH 7.2) for one week. After decalcification, the sternums were processed by standard histological techniques (paraffin-embedding). Five-micrometer sections of sternums were stained by hematoxylin-eosin (H/E) and were evaluated by conventional optical microscopy.

### Bone Marrow Cellularity

The femurs of the control and malnourished mice were removed under aseptic conditions, and the bone marrow cells were flushed from them using Dulbecco’s modified Eagle’s ***medium*** (DMEM) (Vitrocell, Campinas, SP, Brazil) supplemented with 10% fetal calf serum (Vitrocell, Campinas, SP, Brazil). The cells were washed by adding complete medium, centrifuging for 5 minutes at 300 rpm at 24°C, and removing the supernatant.

The mielogram counts were performed by counting cells using a Neubauer chamber (Herka, Berlin, Germany), and the differential cell counts were performed on smears stained with the standard May-Grünwald Giemsa solutions (Sigma Chemical Company, St. Louis, MO, USA). Flow cytometry was used to determine the fraction of the total bone marrow cells that were positively labelled with antibodies against CD117 (cat. no. 553354, Becton Dickinson Pharmingen, San Diego, CA, USA, FITC, clone 2B8) or CD45 (cat. no. 553079, Becton Dickinson Pharmingen, San Diego, CA, USA, FITC, clone 30-F11). The isotype control antibody was FITC-labelled rat immunoglobulin IgG2b kappa FITC (cat. no. 553988, Becton Dickinson Pharmingen, San Diego, CA, USA, FITC, clone A95-1).

### Colony Forming Unit Fibroblastic (CFU-F) Assay

The bone marrow cells from the control and malnourished animals, isolated as described above, were assessed by the CFU-F assay. The CFU-F assay was performed by plating 5×10^5^ cells in 35 mm tissue culture plates (Corning, Tewksbury, MA, USA). The cells were cultured using DMEM (Vitrocell, Campinas, SP, Brazil) supplemented with 10% fetal calf serum (Vitrocell, Campinas, SP, Brazil) for 2 weeks at 37°C in 5% CO_2_ in air. The culture medium was replenished on days 3, 7 and 14 of culture. After 14 days, the developed cell colonies were visualized, and the number of colonies containing more than 50 fibroblastoid cells, denominated CFU-F, was scored using an inverted microscope.

### Isolation of Mesenchymal Stem Cells

The bone marrow cells from the control and malnourished animals were collected as described above. The bone marrow cells were cultured in 25 cm^2^ culture flasks (Corning, Tewksbury, MA, USA) with complete medium at 37°C in 5% CO_2_ in air. The MSCs preferentially attach to the polystyrene surface, and therefore, after 48 h, the suspended non-adherent cells were discarded. Fresh complete medium was added and was replaced every three or four days thereafter. When the cells reached 90% of confluence, the MSC cultures were passaged: the cells were recovered by the addition of a 0.25% trypsin–EDTA (GIBCO Invitrogen, Carlsbad, CA, USA) solution and replated in culture flasks. The cells utilized for experimentation were from passages 3. The cells at passage 3 were confirmed to be mesenchymal stem cells by the pattern of expression of cell surface markers: CD90.2^+^ (cat. no. 553013, Becton Dickinson Pharmingen, San Diego, CA, USA, FITC, clone 30-H12), CD271^+^ (cat. no. ab62122, Abcam, Cambridge, MA, USA, FITC, clone MLR2), Sca1^+^ (cat. no. 557405, Becton Dickinson Pharmingen, San Diego, CA, USA, FITC, clone D7), CD13^+^ (cat no. 558744, Becton Dickinson Pharmingen, San Diego, CA, USA, FITC, clone R3-242), CD34^−^ (cat. no. 5553733, Becton Dickinson Pharmingen, San Diego, CA, USA, FITC, clone RAM34), CD45^−^ (cat. no. 553079, Becton Dickinson Pharmingen, San Diego, CA, USA, FITC, clone 30-F11) and CD14^−^ (cat. no. 553739, Becton Dickinson Pharmingen, San Diego, CA, USA, FITC, clone rmC5-3), and by their capacity for differentiation into osteoblasts, chondrocytes and adipocytes.

### Real- time PCR for PPAR-γ and SREBP in Bone Marrow MSCs

The MSCs from the control and malnourished groups were isolated as described above. The total RNA was obtained from 1×10^6^ MSCs at passage 3 using a RNeasy RNA extraction kit (Qiagen, Germantown, MD, USA) according to the manufactureŕs protocol. The total RNA (3 µg) was reverse-transcribed into cDNA using the high capacity cDNA reverse transcription kit (Applied ***Biosystems, Foster City***, ***CA, USA)***. The cDNA samples were then amplified in the TaqMan universal master mix with optimized concentrations of the primer sets for PPAR-γ (Mm01184322_m1, Applied ***Biosystems, Foster City***, ***CA, USA)*** and SREBP1c (Mm00550338_m1, Applied ***Biosystems, Foster City***, ***CA, USA)***. The internal control used was 18S RNA (Mm03928990_g1, Applied ***Biosystems, Foster City***, ***CA, USA)***. The expression of PPAR-γ and SREBP were evaluated by real-time PCR using StepOnePlus™ (Applied ***Biosystems, Foster City***, ***CA, USA)***. The amplification conditions were as follows: an initial 5 min at 95°C, then 40 cycles of denaturation at 95°C for 10 s and annealing and extension at 60°C for 30 s. The relative quantification of PPAR-γ and SREBP was conducted according to the ΔΔCt method [16].

### Western Blot Analysis

The bone marrow MSCs from the control and malnourished mice were isolated as described above; then, the levels of PPAR-γ and C/EBPα proteins were quantified by the Western blot technique. MSCs were washed three times with sterile cold PBS and lysed with RIPA buffer (0.1% SDS, 1% Igepal CA-630, 1% sodium deoxycholate, 10 mM Tris–HCL, pH 7.5, 150 mM NaCl, 0.5 mM EDTA). To inhibit the activity of proteases and phosphatases, a protease and phosphatase inhibitor cocktail was added (***Sigma***-Aldrich Corp., ***St***. ***Louis***, ***MO, USA***). After centrifugation at 14,000 rpm and 4°C for 15 minutes, the supernatant was collected, mixed with 5× Laemmli buffer (1 M Tris HCl, pH 6.8, 10% 2-mercaptoethanol, 10% SDS, 50% glycerol and 0.01% bromophenol blue) and boiled for 5 minutes. The protein content of the cell homogenates was determined using a BCA Protein Assay kit (***Pierce Biotechnology*** **,** Inc., ***Rockford*** **,** ***IL*** **,** ***USA***), and equal amounts of protein (30 µg per well) were separated on 7.5% SDS-polyacrylamide mini-gels and transferred to Immobilon polyvinylidene difluoride membranes (***Millipore Corporation***, ***Billerica***, ***MA, USA***). After incubation with the appropriate primary antibodies, including 1∶1,000 of anti-PPAR-γ (cat. no. sc7196, ***Santa Cruz Biotechnology***, ***Santa Cruz, CA***, ***USA***) or 1∶500 of anti-C/EBPα (cat. no. sc63486, ***Santa Cruz Biotechnology***, ***Santa Cruz, CA***, ***USA***), the membranes were incubated at room temperature overnight with the primary antibody and for 1 h with a secondary antibody conjugated to horseradish peroxidase (cat. no. DC03L, ***Calbiochem***, ***San Diego,*** ** ***CA, USA***). After three washes with TBST, the immunoreactive bands were visualised using the ECL detection system (Amersham ECL™ Advance Western Blotting Detection Kit, Piscataway, NJ, USA). To standardize and quantify the immunoblots, a digital detection system (IMAGE QUANT™ 400 version 1.0.0, Amersham Biosciences, Pittsburgh, PA, USA) was used. The results were expressed in relation to the intensity for β-actin (1∶20,000 for anti-β-actin, Cell Signaling Technology, Inc., Beverly, MA, USA) as a percentage of the control value.

### Bone Marrow Mesenchymal Stem Cell Culture and SCF, G-CSF, GM-CSF and IL-3 Quantification

Bone marrow MSCs from the control and malnourished mice were isolated as described above and 1×10^6^ MSCs/mL at passage 3 were cultured in DMEM medium (Vitrocell, Campinas, SP, Brazil) supplemented with 10% fetal calf serum (Vitrocell, Campinas, SP, Brazil). These cells were incubated at 37°C in a humidified atmosphere of 5% CO_2_ and on days 3, 7 and 14 of culture, the medium was totally replaced. The medium was collected at the time points of 3, 7 and 14 days of culture and used for the quantification of SCF, G-CSF, GM-CSF and IL-3 by ELISA. The results were measured at each time point and the area under the curve was calculated to evaluate the predictive capability of cytokine production. The tests were performed using a commercially available reagent, Quantikine® M murine (***R&D Systems Inc***, ***Minneapolis, MN***, ***USA***), following the procedures recommended by the manufacturer. The entire procedure was executed under aseptic conditions, and all of the materials used were previously sterilized and were pyrogen-free.

### Adipogenic Differentiation of Bone Marrow Mesenchymal Stem Cells

Bone marrow MSCs from the control and malnourished mice were isolated as described above and 5×10^5^ MSCs at passage 3 were plated in 35 mm tissue plates using DMEM medium supplemented with 10% fetal calf serum and 0.5 µM 1-methyl-3-isobutyl methylxantine (Sigma-Aldrich, St. Louis, MO, USA), 1 µM dexamethasone (Prodome, Campinas, SP, Brazil), 10 µM insulin (Sigma-Aldrich, St. Louis, MO, USA; I0516), and 200 µM indomethacin (Sigma-Aldrich, St. Louis, MO, USA) (Romanov et al., 2003). After 1 week, the cultures were fixed in a 10% formaldehyde solution (Sigma) for 1 hour, washed with isopropanol (Sigma) and stained with Oil Red O solution (in 60% isopropanol) for 5 minutes followed by five washes with PBS and then counterstained with the standard May-Grunwald Giemsa solutions (Sigma) and the number of adipocytes was quantified by microscopy. At the same time, cultures stained with Oil Red O without counterstaining were destained by 100% isopropanol for 15 minutes, and the optical density (O.D.) of the solution was measured at 540 nm in a microplate reader.

### Statistical Analysis

The results are expressed as the mean with the standard deviation. Differences between groups were examined using Student´s *t* test (p≤0.05). The cytokine values were analyzed from the area under the curve, and the results were examined using Student´s *t* test (p≤0.05). All tests were performed using the computer software Graphpad Prism^®^.

## Results

### Diets, Food Consumption, Protein Consumption, Body Weight Variation, Plasma Protein, Pre-albumin, and Albumin Concentrations

The animals in the malnourished group consumed the same quantity of food and calories did as the controls, but their ingestion of protein was reduced because the hypoproteic diet had a lower protein content. The animals from the malnourished group exhibited a significant loss of body weight, of approximately 20% of their initial weight, and a significant decrease in total serum protein, albumin and pre-albumin concentrations (**Table 1**).

**Table 1:** Protein intake, body weight variation, blood cell count, and concentrations of plasma protein, albumin, and pre-albumin of control and malnourished mice.

Groups	Control (n = 12)	Malnourished (n = 12)
**Diet consumption (g/day/animal)**	6.14±0.78	6.04±0.70
**Protein consumption (g/day/animal)**	0.73±0.09	0.12±0.01***
**Body weight variation (%)**	6.33±1.3	−17.7±1.3*
**Plasma protein (g/dL)**	6.7±0.44	4.0±0.61*
**Albumin (g/dL)**	3.2±0.61	2.1±0.44*
**Pre-albumin (mg/dL)**	10.9±1.9	2.97±1.1*
**Erythrocytes (x 10^6^/mm3)**	8.77±0.29	6.99±0.37*
**Hemoglobin (g/dL)**	14.9±0.33	11.9±0.47*
**Hematocrit (%)**	44.5±0.77	36.9±0.99*
**Leukocytes (/mm^3^)**	2790±490	1008±189*
**Neutrophils (/mm^3^)**	189±2.7	33.7±1.9*
**Eosinophils (/mm^3^)**	9.9±0.11	1.4±0.03*
**Lymphocytes (/mm^3^)**	2425±238	974±111*
**Monocytes (/mm^3^)**	59±6.1	18.1±1.9*

The results, expressed as the means with standard deviations, are presented for protein consumption, change in body weight, serum protein, albumin, and pre-albumin concentrations, number of erythrocytes, hemoglobin concentration, hematocrit, number of reticulocytes, and total number of leukocytes, neutrophils, lymphocytes, and monocytes of control and malnourished animals. The number in parentheses denotes the total number of animals used in the experiment.

* (p ≤ 0.05) and ***(p ≤ 0.001) indicates where there was a significant difference between the control group and the malnourished group.

### Blood, Spleen and Bone Marrow Cellularity

The hemogram of the malnourished animals showed anemia and leucopoenia with a significant reduction in neutrophils. Significant morphological differences in erythrocytes were not found (**Table 1**).

The spleen cellularity showed a significant reduction in malnourished animals in comparison to the control animals. The mielogram of the malnourished animals showed a significant reduction of the bone marrow cellularity with a significant reduction in polymorphonuclear cells, macrophages, and erythroblasts (**Table 2**).

**Table 2:** Bone marrow and spleen cell count of control and malnourished animals.

Groups	Cells (x10^6^/mL)			
	Bone Marrow		Spleen	
	Control (n = 12)	Malnourished (n = 12)	Control (n = 12)	Malnourished (n = 12)
**Total Cells**	10.01±2.20	6.16±1.11**	11.24±1.78	8.08±1.01***
**Blast cells**	0.61±0.08	0.25±0.03**	0.37±0.11	0.24±0.11*
**Neutrophils: promyelocytic and myelocytic cells**	0.82±0.09	0.52±0.02*	0.43±0.19	0.27±0.21
**Neutrophils: band cells**	1.71±0.18	1.14±0.13*	0.29±0.11	0.19±0.07*
**Neutrophils: segmented**	2.22±0.28	1.45±0.14**	0.63±0.16	0.30±0.13***
**Eosinophils**	0.20±0.05	0.07±0.01*	0.06±0.06	0.02±0.04
**Erythroblasts**	1.52±0.18	0.22±0.05***	1.78±1.04	0.87±0.25***
**Lymphocytes**	2.41±0.21	1.90±0.15*	7.29±1.46	5.91±0.93*
**Macrophages**	0.17±0.02	0.01±0.02*	0.09±0.11	0.07±0.06

The results are expressed as the means with standard deviation for the total and differential cell counts of bone marrow and spleen cells of the control and malnourished animals. The number in parentheses denotes the total number of animals used in the experiment.

* (p ≤ 0.05), **(p ≤ 0.01) and ***(p ≤ 0.001) indicates where there was a significant difference between the control group and the malnourished group.

### Bone Marrow Histology

**Figure 1:**
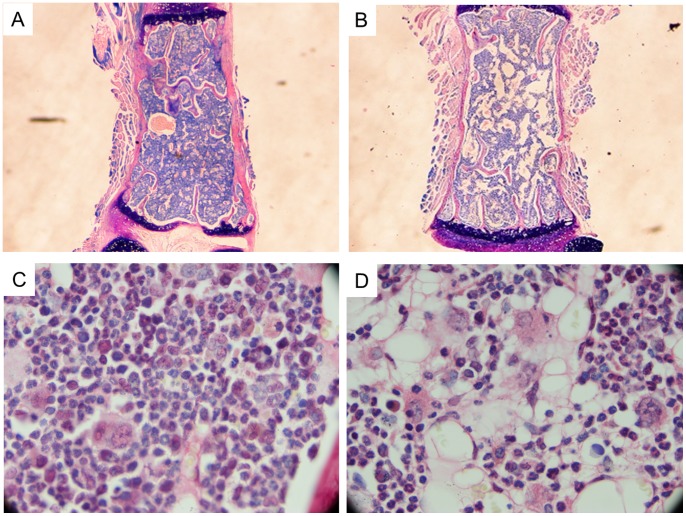
Sections of bone and marrow from control and malnourished animals. (A) Bone marrow biopsy section from a control animal showing normal cellularity with heterogeneous populations of cells at different stages of maturation. Embedded in paraffin (HE stain, ×4). (**B**) Bone marrow biopsy section from a malnourished animal showing severe hypocellularity. Embedded in paraffin (HE stain, ×4). (**C**) Bone marrow biopsy section from a control animal showing normal cellularity. Embedded in paraffin (HE stain, ×1000). (**D**) Bone marrow biopsy section from a malnourished animal showing hypocellularity and adipocytes increase. Embedded in paraffin (HE stain, ×1000). Sections are representative of control (n = 6) or malnourished group (n = 6).

The malnourished group presented with shrinkage of the marrow hematopoietic space leading to a hypocellular bone marrow with depletion in all hematopoietic lineages as well as the presence of interstitial dilated areas with medullary lakes determinated by dilated vessel lumens sinus and adipocyte-rich marrow (**Figure 1B and 1D**), whereas the control group presented a normocellular bone marrow with a predominantly granulocytic series (**Figure 1A and 1C**).

### Bone Marrow CD45^+^ and CD117^+^ Cells Quantification

**Figure 2:**
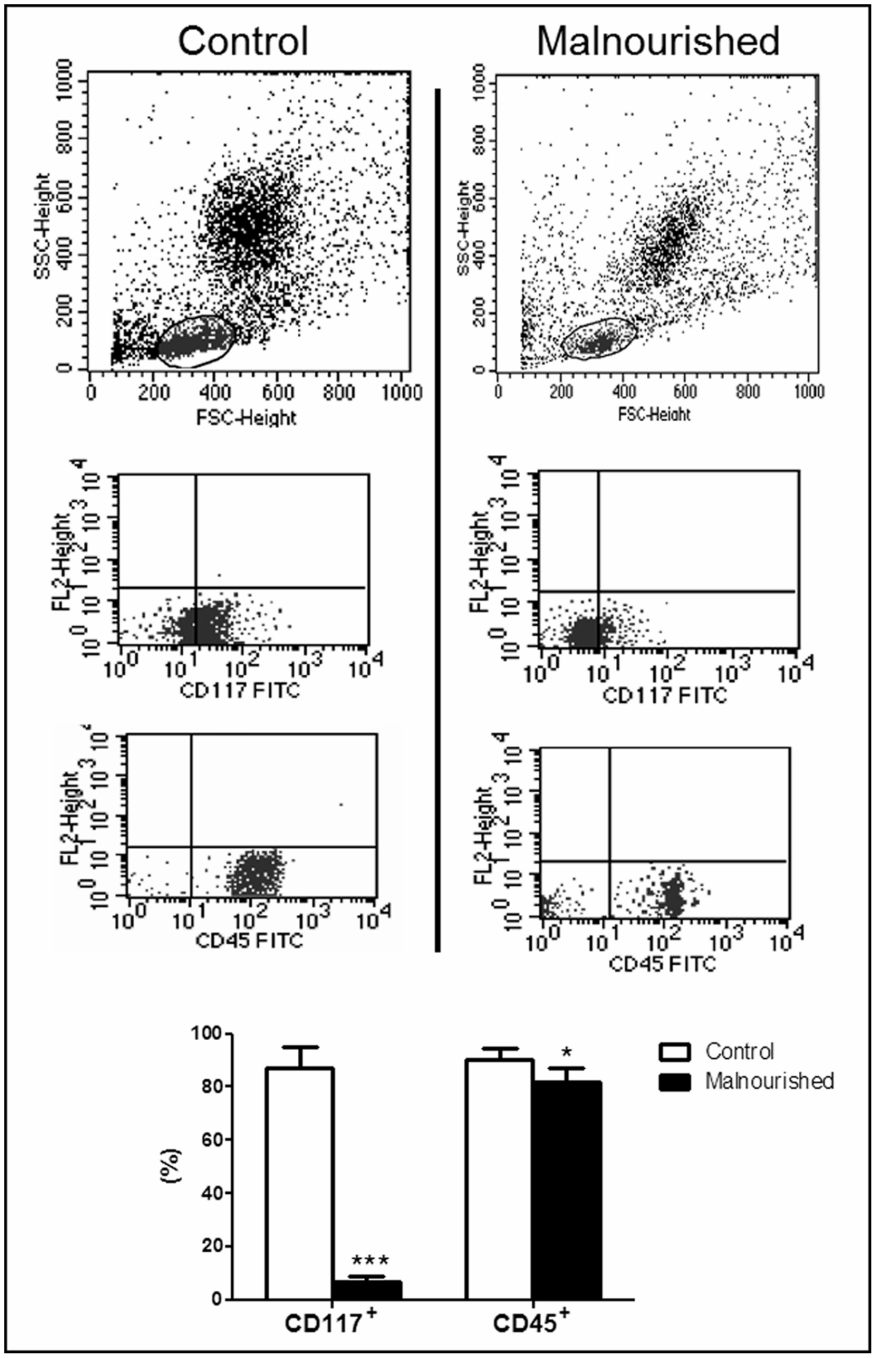
Flow cytometry analyses of the bone marrow of control and malnourished mice. The selection of gates, R1, for the cell populations that were not labeled with fluorochromes are indicated (SSC in the y-axis vs. FSC in the x-axis). Immunophenotypical bone marrow analysis was carried out in gate R1 for CD117 and CD45 expression. The graphical results are expressed as the means with standard deviations for CD117^+^ and CD45^+^ expression by control (n = 6) and malnourished (n = 6) mice. The number in brackets denotes the number of animals used in the experiment. *(p ≤ 0.05) and ***(p ≤ 0.001) indicate where there was a significant difference between the control group and the malnourished group.

The bone marrow CD45^+^ and CD117^+^ cell quantification showed a reduced number of CD45^+^ and CD117^+^ cells in malnourished animals when compared to control animals **(** **Figure 2** **).**

### MSCs Isolation and Colony Forming Unit Fibroblast (CFU-F) Assay

Our flow cytometry results showed that the cells isolated by the method classically described by Friedenstein et al. [17], [18] and popularized by Caplan [19] that utilizes the physical property of adherence to plastic were a population that stained positive for CD90, CD271, CD49e, CD13 and Sca-1 and were negatively labeled for CD34, CD45 and CD14, with no contamination by hematopoietic cells (data not show). The cultures showed a heterogeneous morphology containing cells ranging from tapered and pointed fibroblast cells, to broad, polygonal cells, as described by Javazon et al. [20]. The colony-forming unit-fibroblast (CFU-F) assay showed a morphologically heterogeneous population in both groups (data not show). The quantification of CFU-F were performed on day 14 of culture and our results did not show differences between the malnourished (43.2±2.5 CFU-Fs) and control (44.8±1.8 CFU-Fs) animals.

### Real- time PCR for PPAR-γ and SREBP in Bone Marrow MSCs

**Figure 3:**
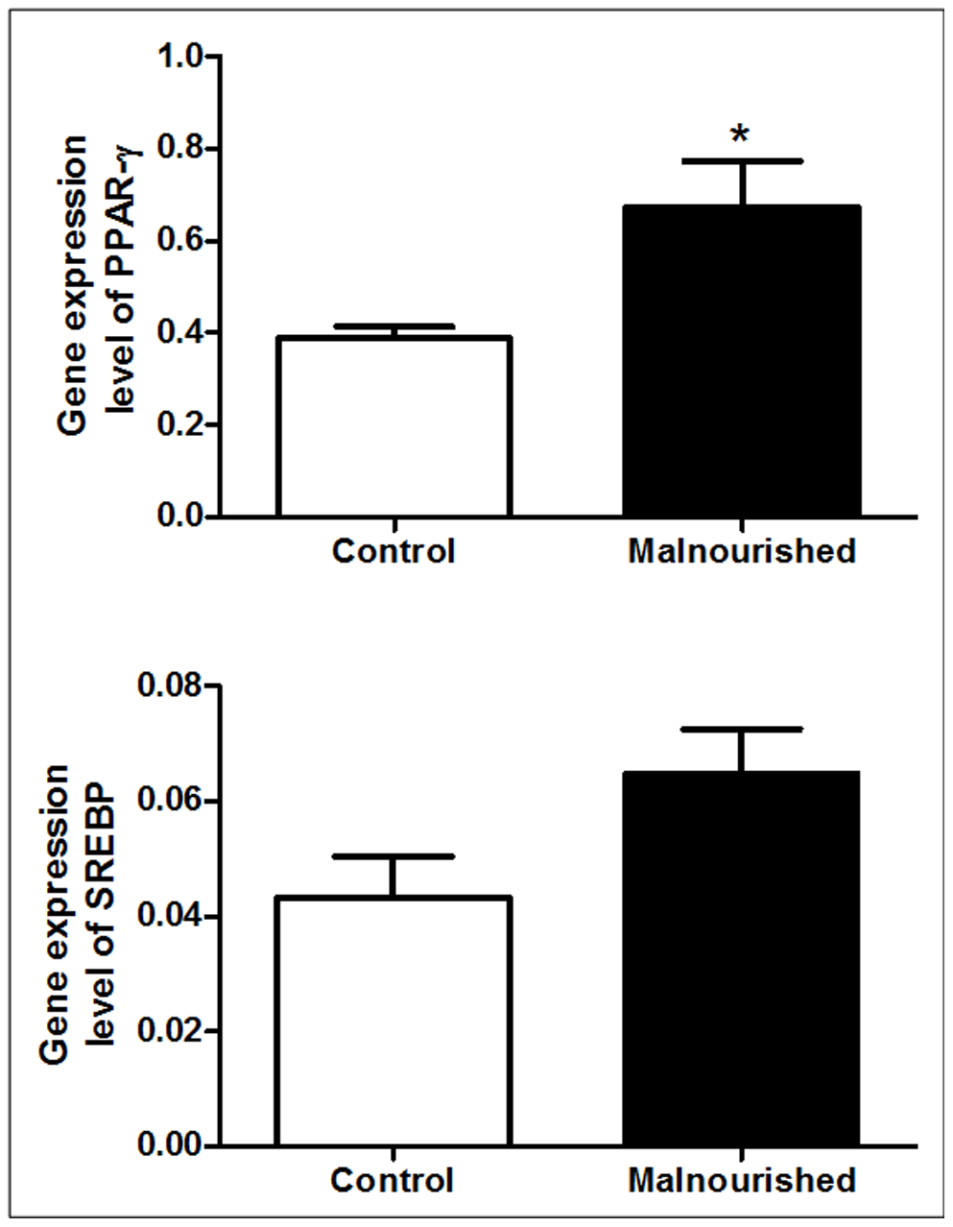
The results, expressed as the means with standard deviations, for the relative gene expression of PPAR-γ and SREBP in MSCs from control (n = 6) and malnourished animals (n = 6). Data were normalized to 18S. The number in brackets denotes the number of animals used in the experiment. *(p ≤ 0.05) indicates where there was a significant difference between the control group and the malnourished group.

The gene expression of PPAR-γ in the MSC from malnourished animals was increased when compared to control animals. PPAR-γ is a transcription factor and considered the master regulator of cells undergoing adipogenic differentiation. The levels of gene expression of SREBP were not significantly different between the groups, although a tendency toward increased expression was observed in malnourished animals when compared to control animals (**Figure 3**).

### Expression of PPAR-γ and C/EBPα in MSCs

**Figure 4:**
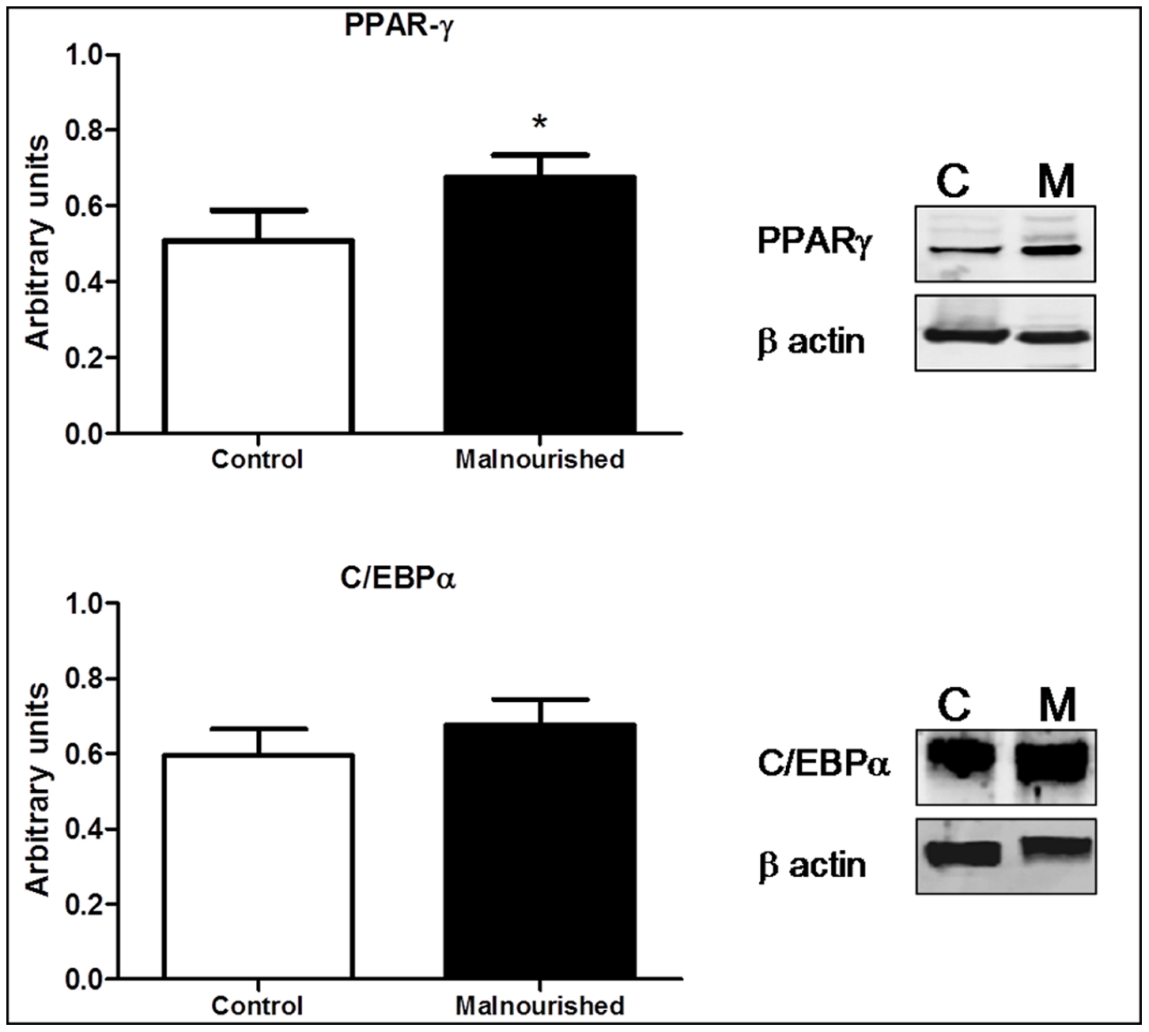
The results, expressed as the means with standard deviations, for the expression levels of PPAR-γ and C/EBPα in MSCs from control (n = 6) and malnourished (n = 6) animals as determined by Western blot analysis. The Western blot image was representative of six independent experiments with similar results. After densitometric analysis, results for PPAR-γ and C/EBPα protein expression were normalized to β-actin values. The number in brackets denotes the number of animals used in the experiment. *(p ≤ 0.05) indicates where there was a significant difference between the control group and the malnourished group.

The transcription factors related to adipogenesis, PPAR-γ and C/EBPα were evaluated in isolated bone marrow MSCs by Western blotting (**Figure 4**). Our results demonstrated an increased expression of PPAR-γ in MSCs from malnourished animals when compared to control animals. We did not observe a significant difference in the expression of C/EBPα in MSCs from malnourished and control animals, although the MSCs from malnourished animals showed a tendency to have higher levels. These results indicate that protein malnutrition can change the balance of adipogenesis of MSCs from mice bone marrow MSCs.

### Determination of SCF, G-CSF, GM-CSF and IL-3 Production in vitro

**Figure 5:**
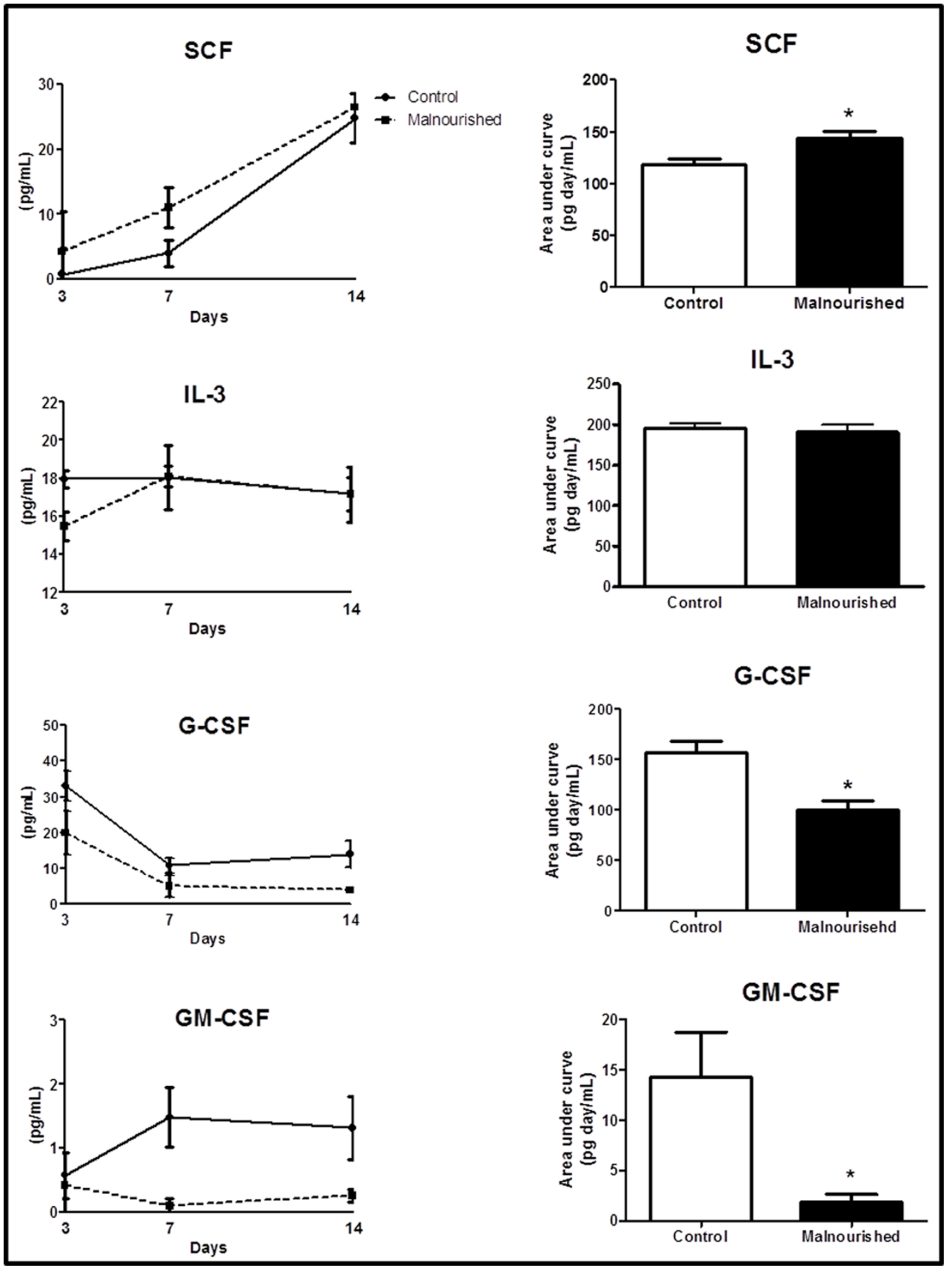
The results are expressed as the means with standard deviations and the area-under-the-curve of the production kinetics of SCF, IL-3, G-CSF and GM-CSF in MSC from control (n = 6) and malnourished (n = 6) animals cultured for 14 days. The number between the brackets denotes the number of samples evaluated per group in each timepoint studied. *(p ≤ 0.05) indicates where there was a significant difference between the control group and the malnourished group.

The supernatant was collected from the cultured MSCs for measurements of the cytokines produced by these cells. The results presented assessing the kinetics of production of SCF show that MSCs from the malnourished animals had a higher capacity to produce SCF and a lower capacity to produce G-CSF and GM-CSF when compared to control animals. In relation to IL-3 production, we did not observe a difference between the groups (**Figure 5**).

### Adipogenic Differentiation of MSCs

**Figure 6:**
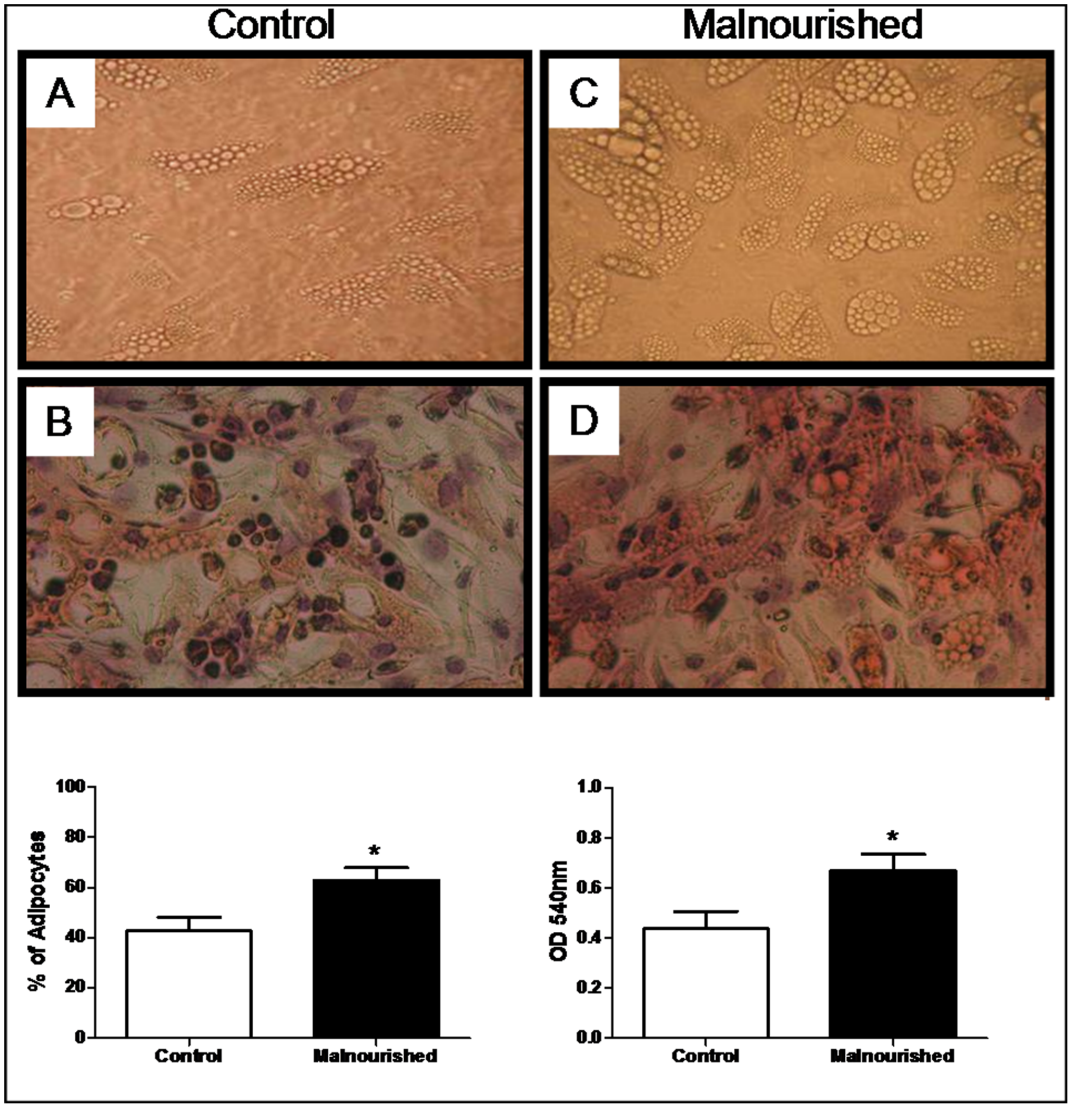
MSCs cultures from control and malnourished animals induced to adipogenic differentiation. (A) Cell culture morphology of differentiated MSCs from control animals cultured in adipogenic media (x 100); (B) Oil red O positive cells on day 14 after adipogenic differentiation of control MSCs. The red parts are lipid-rich vacuoles, the blue ones are nuclei counterstained with the standard May-Grunwald Giemsa solutions (x 100); (C) Cell culture morphology of differentiated MSCs from malnourished animals cultured in adipogenic medium (x 100); (B) Oil red O positive cells on day 14 after adipogenic differentiation of malnourished MSCs. The red parts are lipid-rich vacuoles, the blue ones are nuclei counterstained with the standard May-Grunwald Giemsa solutions (x 100). Representative pictures from control (n = 6) and malnourished groups (n = 6). The results showing the graphic percentage of quantified adipocytes and the graphic Oil red quantification from control (n = 6) and malnourished (n = 6) animals are expressed as as the means with standard deviations. The number in brackets denotes the number of animals used in the experiment. *(p ≤ 0.05) indicates where there was a significant difference between the control group and the malnourished group.

Bone marrow MSCs from both groups were induced to differentiate into adipocytes. It was observed that mesenchymal stem cells obtained from the bone marrow of mice have the capacity to differentiate into adipocytes, and this capacity, after 14 days of culture, was higher in cells obtained from malnourished animals when compared to control animals (Figure 6).

## Discussion

This study indicates that protein malnutrition induced central and peripheral leukopenia, and these results are, in part, due to a hematopoiesis compromise resulting from changes in the hematopoietic cells as well as in the bone marrow MSCs.

We developed a protein malnourished model where animals induced to undernutrition consumed a reduced amount of protein. Because the animals from the control and malnourished groups consumed the same quantity of food, we established protein malnutrition when the control group received a diet that contained adequate protein while the experimental group received a protein-deficient diet. The minimum daily amounts of nutrients other than protein were ingested by the animals of the malnourished group. The animals from the malnourished group showed a marked loss, approximately 20%, of body weight with a reduction of protein, albumin and pre-albumin serum levels.

Our results showed leukopenia with neutropenia associated with spleen and bone marrow hypoplasia. The first reports on blood leukopenia in protein malnutrition were made by Kornberg et al. [21] and Aschkenasy [22]. Even though the leukocyte response is variable, there is evidence that in situations in which malnutrition is not accompanied by other diseases, leukopenia is always present [3], [23], [24], [25]. The bone marrow hypocellularity found in the current study was also reported by Flo et al. [26], Olmos [27] and our group [1], [2], [3]. This bone marrow hypoplasia is combined with a reduction of CD45^+^ and CD117^+^ cells. These results are in agreement with other studies showing that deletion mutation of CD45 reduced the number of primitive hematopoietic stem cells [28] and that the lack of CD117 receptors is associated with a quiescent and incapable hematopoietic reconstitution [29], which is in accordance with previous results from our group which showed that protein malnutrition produces qualitative and quantitative alterations in hematopoiesis that are caused by a structural impairment of the bone marrow stroma and alterations in the hematopoietic progenitors’ cell cycle with a higher number of cells in the G0/G1 phase [2].

MSCs are multipotent precursors and in the bone marrow give rise to cells that constitute the bone marrow stroma; our results showed that MSCs were isolated and that no contamination with hematopoietic cells was observed. The CFU-F assay did not show differences between the groups as well as the production of IL-3, a cytokine capable of stimulating proliferation, differentiation, and survival of hematopoietic stem cells [29]. However, our results showed that MSCs from malnourished animals showed a reduced capacity to produce G-CSF and GM-CSF, whereas the production of SCF was increased. The literature reports [30], [31], [32], [33] that the lack of SCF production by accessory cells or the absence of receptors for this growth factor in hematopoietic stem cells leads to a hematopoietic failure, showing that MSCs have an important function in the regulation of the hematopoietic niche [34], [35]. The commitment to the expression of CD117^+^ and CD45^+^ hematopoietic cells in the malnourished animals could be explained by an increased production of SCF and a decreased production of G-CSF and GM-CSF by the MSCs. Evidence shows that MSCs are able to support the expansion and differentiation of the hematopoietic stem cells due to their capability to produce cytokines [34], [36], [37]. Thinking of this capability, we hypothesized that the increased production of SCF could be related primarily in maintenance of the hematopoietic stem/progenitor cell pool, once this cell pool is compromised by protein malnutrition states and cells are arrested in G0/G1 phases of the cell cycle [2]. Therefore, the increase of SCF observed in MSCs from the malnourished animals could be because the reduced expression of CD117 receptors in hematopoietic cells and the increase in SCF would have the effect to try to stimulate the proliferation of hematopoietic stem and progenitor cells.

Concerning the reduced production of G-CSF and GM-CSF by MSCs from the malnourished animals, this can also be explained by the reduced stem/progenitor cell pool because G-CSF and GM-CSF are growth factors that act on proliferation but primarily on the granulocytic and monocytic differentiation [38], and their reduced production could be responsible for the commitment to granulopoiesis commonly observed in protein malnutrition states [1], [2].

Protein malnutrition produces both qualitative and quantitative alterations of both the stromal components and the extracellular matrix of bone marrow, inducing a decrease in hematopoiesis [1], [2]. Evidence shows bone marrow hypoplasia in protein malnutrition states, and our results are in agreement with these findings, where bone marrow hypoplasia was observed in the malnourished animals and was associated with this hypoplasia. We hypothesized that an increase in the adipogenic bone marrow state could in part be an explanation for the bone marrow failure in protein malnutrition states because the increase in adipocytes in the bone marrow microenviroment has the capability to negatively modulate hematopoiesis, as previously reported by Naveiras [9].

To test the hypothesis that increased marrow adiposity may be a consequence of bone marrow hypoplasia, we isolated MSCs from the bone marrow and differentiated these cells into adipocytes *in vitro.* Our results showed that the MSCs isolated from malnourished animals showed a higher capability to differentiate into adipocytes when cultured in a specific medium containing an adipogenic cocktail.

The activation of certain transcription factors is essential for cellular commitment to a particular lineage. Adipogenic differentiation is accompanied not only by alterations in cell morphology but also by transcriptional activation, including that of PPAR-γ and C/EBPα [11], [39], as well as an increase in the sterol regulatory element-binding protein (SREBP) that has been identified as an important transcription factor regulating lipogenesis in multiple tissues [40].

PPARγ is the master transcription factor that plays a requisite role in the adipocyte differentiation process and which alone can initiate adipogenesis [11], [12], [41]. Overexpression of PPARγ in fibroblast cell lines was shown to be sufficient to initiate adipocytogenesis, whereas cells from mice lacking PPARγ were unable to differentiate into adipocytes [12]. This evidence supports our findings where MSCs isolated from malnourished animals have higher expression of PPARγ and, as reported above, a greater capability to differentiate into adipocytes when cultured in an adipogenic inducing medium. Moreover, the literature reports that bone marrow-derived adipocytes reduce the expansion of hematopoietic cells in a co-culture system [9], indicating that adipocytes release diffusible inhibitors of hematopoiesis.

The highlight of these findings is that we showed an association between the increase in bone marrow hypoplasia and the marrow’s capacities for cytokine production that resulted in an altered hematopoiesis. These associated results permit the conclusion that the alterations of the bone marrow microenviroment resulting from protein malnutrition could, in part, explain its ***hematopoietic failure*** *.*
